# Competition between Methanogens and Acetogens in Biocathodes: A Comparison between Potentiostatic and Galvanostatic Control

**DOI:** 10.3390/ijms18010204

**Published:** 2017-01-19

**Authors:** Sam D. Molenaar, Pradip Saha, Annemerel R. Mol, Tom H. J. A. Sleutels, Annemiek ter Heijne, Cees J. N. Buisman

**Affiliations:** 1 Wetsus, European Centre of Excellence for Sustainable Water Technology, Oostergoweg 9, 8911 MA Leeuwarden, The Netherlands; annemerel.mol@wur.nl (A.R.M.); tom.sleutels@wetsus.nl (T.H.J.A.S.); cees.buisman@wetsus.nl (C.J.N.B.); 2 Department of Environmental Technology, Wageningen University, Bornse Weilanden 9, 6708 WG Wageningen, The Netherlands; Pradip.Saha@wur.nl (P.S.); annemiek.terheijne@wur.nl (A.t.H.)

**Keywords:** biocathode, acetate, current density, microbial electrosynthesis (MES), bioelectrochemical systems (BES), kinetics, thermodynamics, methanogen, acetogen, competition

## Abstract

Microbial electrosynthesis is a useful form of technology for the renewable production of organic commodities from biologically catalyzed reduction of CO_2_. However, for the technology to become applicable, process selectivity, stability and efficiency need strong improvement. Here we report on the effect of different electrochemical control modes (potentiostatic/galvanostatic) on both the start-up characteristics and steady-state performance of biocathodes using a non-enriched mixed-culture inoculum. Based on our results, it seems that kinetic differences exist between the two dominant functional microbial groups (i.e., homoacetogens and methanogens) and that by applying different current densities, these differences may be exploited to steer product selectivity and reactor performance.

## 1. Introduction

The use of bioelectrochemical systems (BES) for the production of organic compounds such as alcohols or short- to medium-chain fatty acids through microbial electrosynthesis (MES) holds potential [1]. By exploiting the capabilities of microorganisms to metabolize using electrodes as electron donors—A feature that is found to be widespread amongst most diverse groups of microorganisms [2]—A variety of organic compounds can be synthesized in mild operational conditions [3,4,5]. As such, these systems provide an environmentally friendly means for producing useful chemicals using cheap and available substrates (carbon dioxide and water), while avoiding the requirement of scarce metal catalysts in the cathode, as would be the case in most conventional electrosynthetic processes.

While the products potentially formed in MES are found to be diverse, the main scientific efforts in this field have so far focused on the specific production of acetate by the reduction of carbon dioxide [6,7,8,9,10,11]. There are two general considerations at the basis of this product choice: (1) specificity; and (2) reaction rate. With the standard reduction potential of acetate from CO_2_ being the highest amongst all carboxylates (and considerably higher than that of formate), the potential window within which the cathodic production of acetate can take place shows minimal overlap with respect to most alternative CO_2_ reduction products. This provides a thermodynamic advantage regarding the specificity towards the production of acetate. In addition, with the number of electrons (eight) still being considerably low compared to higher reduction products like butyrate, reaction rates are not severely kinetically limited, and production of acetate at high specificity (up to 100%), and in some cases at high rates (up to 66 kg·m^−3^·day^−1^), has been achieved in biocathodes [7].

Troubling to the specific and efficient production of acetate in BES is the production of methane by reduction of carbon dioxide, with a very comparable standard reduction potential (Table 1).

As follows from Table 1, given its standard potential methanogenesis may always be regarded as competitive to acetate production from a thermodynamic perspective, with methane either produced from H_2_ and CO_2_ instead of acetate (in case higher overpotentials are used), or produced acetate being consumed through the disproportionation reaction used in acetoclastic methanogenesis.

**Figure 1:**
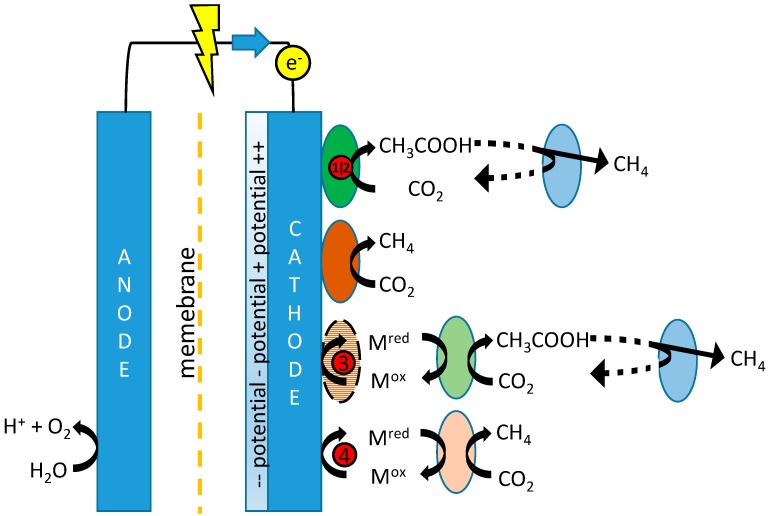
Different metabolic pathways in a mixed-culture biocathode as proposed in different studies. A distinction can be made between direct electron transfer [18], in which microbes are directly attached to the electrode surface, thus allowing electrons to reach the intracellular environment by means of electrical conductance, and the use of a redox active chemical mediator (M_ox_/M_red_) in the case of indirect electron transfer. Several studies have suggested a combination, in which direct electron transfer leads to the biocatalysed reduction of a mediator compound, which then functions as electron donor for suspended cultures [7,8]. Regardless of whether initial electron transfer takes place directly or indirectly, close competition between methanogens (**brown**) and acetogens (**green**) will exist for available electrons. Regarding the composition of a possible mediator, although in theory any kind of mediator could be shuttling electrons back and forth between electrode and microbe as long as its reversible redox potential is adequate, in practice this role is most likely fulfilled by hydrogen, although formate has been hypothesized to play a role as secondary mediator as well [1]. Any formed acetate can be further oxidized acetoclastically to form CO_2_ and methane (**blue microbe**). The red-circled numbers refer to the potential levels tested in this study, aimed at establishing indicated pathways and are further defined in the materials and methods section.

While the production of methane at biocathodes may form a target in itself, with applications in, for instance, biogas upgrading or the conversion of organic waste into natural gas [13,14], the distinct uses and higher economical value of acetate make taking control over specificity in biocathodes with respect to production of both compounds worthwhile. Substantial scientific efforts to this end have been made using diverse methodologies [3,8,10,15,16,17]. The findings are summarized in Figure 1, which depicts the possible mechanisms for production of both acetate and methane in MES, including references proposing these mechanisms.

For the production of acetate, two strategies to prevent methanogenesis may be distinguished: (1) pre-enrichment of inoculum or the use of pure cultures while working with aseptic conditions to prevent contamination of the systems with methanogens, thus effectively keeping out competitive microorganisms [17]; and (2) chemical inhibition of methanogenesis [7]. Although these techniques have been shown successful to some extent, none of these practices alone provide a means for continuous, larger-scale and long-term septic reactor operation. The use of pure cultures or enriched inocula is prone to contamination and use of chemical inhibitors is unattractive both from economical as well as environmental perspective. Additionally, methanogens may develop immunity towards the inhibiting compounds used, rendering them less effective over time [19].

The need thus exists for a more integrated approach towards the selective and stable production of acetate, which takes into account (1) structural differences between microbial groups; (2) differences in thermodynamics; and (3) kinetics associated with the proposed pathways in Figure 1 to affect product specificity. Based on these aspects, reactor operational parameters need to be found at which biomass retention time for acetogens is higher than for methanogens, and/or keeping growth rates (and thus metabolic activity) of acetogens higher than those of methanogens.

Tangent to the considerations of fast kinetics and long acetogenic biomass retention is the question as to whether biocathodes are best operated using potentiostatic or galvanostatic control. A constant potential may be set to narrow down the possibly involved electron transfer mechanisms (Figure 1) and may lead to the formation of a more energy efficient microbial community. Constant current, on the other hand, allows for better control over production rates and possibly selectivity as it is less sensitive or dependent on (local) changes in e.g., pH, and the electron flux can be controlled to match stoichiometrically with the rate of CO_2_ supply.

In this study, the performance of graphite felt electrodes was tested for the cathodic production of acetate using a mixed culture inoculum from AD sludge, using continuously operated reactors. The effect of using both potentiostatic and galvanostatic control on reactor performance was analysed and compared. By using a mixed culture inoculum, the presence of both acetogens and methanogens was assured. Different cathode potentials were tested during potentiostatic experiments, and acetate production was compared to current controlled experiments, in which reducing equivalents were provided at constant rate. Results were interpreted to provide more insight into the mechanisms at play in biocathodes, and the consequential perspectives on future reactor optimization.

## 2. Results

### 2.1. Potential Controlled Experiments

**Figure 2:**
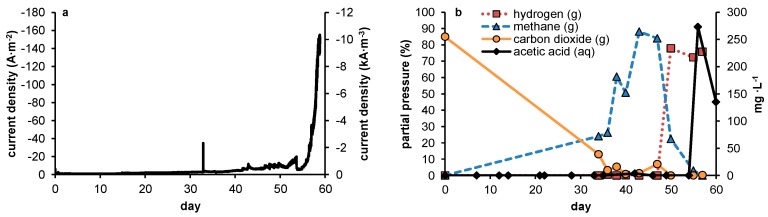
(**a**) current plot of the −900 mV vs. Ag/AgCl controlled biocathode throughout 57 days of operation; (**b**) Headspace composition throughout the experiments showed gradually increasing methane partial pressures, accompanied by decreasing levels of carbon dioxide. In the last phase of the experiment, hydrogen replaced all other gases and subsequently acetate formation took place.

Four cathode electrode potentials were selected based on thermodynamics, and this is further explained in the Materials and Methods section. The three cells with the least negative cathode potential (−560, −630 and −700 mV) showed neither current nor acetate production during 34 days of operation. For this reason, these experiments were then ended and their results are not further reported on here. The fourth, most negative cathode potential (−900 mV) showed increasing current during this period, and concomitantly measurable amounts of reduction products were produced. The development throughout the experiment of current, acetate concentration and partial pressures of methane, carbon dioxide and hydrogen as measured in the headspace gas composition of this last cell are displayed in Figure 2.

As can be seen in Figure 2a, during the first 47 days no reduction products other than methane were detected, with methane taking an increasingly large share in the headspace composition and reducing the partial pressure of CO_2_ as measured in the headspace from 85% initially to 5%–10% by day 47. The cathodic current continued to increase steadily up to day 50, at which point a fast decline in methane in the headspace was observed while the amount of hydrogen in the headspace increased dramatically. Directly following this shift in reduction products, the current continued to increase slightly until day 54, accompanied by further decreasing methane partial pressure in the headspace. At day 54, after an initial drop in current density to approximately 7 A·m^−2^, an exponential growth in current started, with a resulting current of 350 mA (157 A·m^−2^ projected surface area) after 57 days. At this point, the experiment was ended because the applied voltage increased above the maximum 10 V output of the potentiostat. Hydrogen had fully replaced the previously formed methane in the headspace and an acetate concentration of 270 mg·L^−1^ was found in the electrolyte. Despite the low pressure of CO_2_ remaining in the headspace at this point, the system was continuously fed with influent containing 11 mM total dissolved inorganic carbon (DIC). This influent concentration provided sufficient carbon for the observed concentration of acetate in Figure 2b: 270 mg·L^−1^ acetic acid corresponds to 4.57 mM, and would thus require at least 9.15 mM DIC. With no longer potential or current applied to the system, acetate concentrations roughly halved at day 60, after which no more measurements were taken.

The increase in current production, followed by a decline in the production of methane, was interpreted as follows: first (day 0 to 54), with pH levels stable at pH 6.1 throughout operation of the reactor, a steady increase in production of both current and methane suggested the progressive growth of a (presumably but not necessarily hydrogenotrophic) methanogenic community. During this period, the increase in current resulted from the increasing biological activity either by means of hydrogen-scavenging, or through the development of other, higher potential redox shuttling towards methane. Depletion of CO_2_ started to hamper production of methane from day 47 onwards, resulting in a switch towards the net production of hydrogen which then became the dominant reduction product. Finally, the resulting elevated hydrogen partial pressure led to a production of acetate through homoacetogenesis, which until that point had been outcompeted by hydrogenotrophic methanogenesis. Concomitantly with the onset of acetogenesis, current production entered an exponential phase (day 54 to 57).

To summarize, in the four potential controlled experiments, no reduction reactions occurred at potentials above or near the equilibrium potential for hydrogen formation. Therefore, although based on the limiting period of testing the possibility of reduction of CO_2_ at low overpotential—thus by more direct means—Cannot conclusively be excluded, it seems to be unfeasible. Only for the highest chosen overpotential was formation of methane and later hydrogen and acetate observed.

### 2.2. Current Controlled Experiments

**Figure 3:**
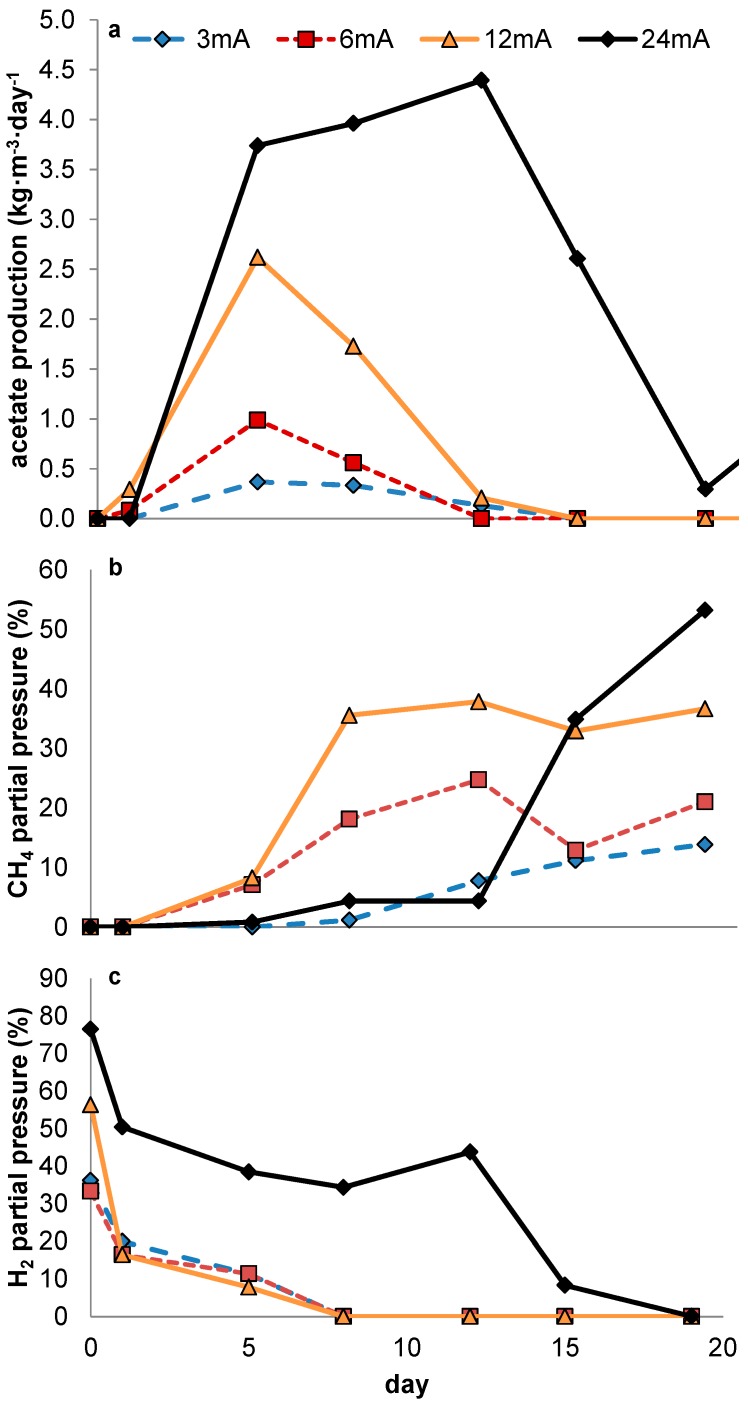
Results obtained from four biocathodes controlled at different current densities (3, 6, 12 and 24 mA corresponding to 1.3, 2.6, 5 and 11 A·m^−2^, respectively): (**a**) Observed acetate production rates as calculated from effluent concentration measurements show a clear effect of current density; while (**b**) methane partial pressures; and (**c**) hydrogen partial pressures as measured by head space gas analysis indicate progressive loss of electrons to methane as hydrogen partial pressure decreases.

The potential controlled experiments suggested hydrogen formation to be required for CO_2_ reduction under the chosen reactor conditions. Moreover, with the observation of acetate production only established at higher current densities, the effect of current density was further investigated, controlling current at four different levels (1.3, 2.6, 5 and 11 A·m^−2^). As can be seen in Figure 3, all current-controlled cells showed production of acetate directly after start-up. In addition to the formed acetate, hydrogen was also detected in the off-gas of the reactor, even at the lowest chosen current densities.

The production of acetate with all four current densities tested showed to be unstable, reaching peak levels after 5–13 days, followed by a decline. During acetate peak production, Coulombic efficiencies in all reactors ranged from 60% to 100% (more detailed information can be found in Figure S2. After 19 days, none of the reactors produced measurable amounts of acetate or hydrogen, and it was assumed current was fully diverted towards the production of methane, with headspace methane partial pressures ranging from 16% for the lowest current to 55% for the highest current tested. At this point it should be remarked that the partial pressures reported do not directly relate to gas production. The gas flow measurements therefore required were unfortunately lacking due to the continuous design of the reactors resulting in inaccurate measuring of produced gas flow. Judging from the available data, an increase in partial pressure certainly indicates increased production of a compound qualitatively. However, an apparently stagnant or slightly changing partial pressure does not necessarily indicate unchanged production rates as small changes in partial pressure can already imply a large change in flux due to the large buffering capacity of CO_2_, and through increased losses through transmembrane transport.

Continuous pH measurements showed rather constant levels throughout all tested current densities, and were not considered of relevance to the observed outcome (data in Figure S1).

Apart from reaching different peak production levels for acetate, the different currents tested showed variation in the time elapsed before peak acetate production occurred, with the highest current showing a later peak compared to lower currents. For all cells tested, headspace analysis showed increasing methane partial pressures coinciding with declining acetate levels, suggesting the decrease in acetate production was due to increasing activity of methanogens. The question then arises as to whether the observed decline in acetate production was due to competition of methanogens for hydrogen, or that—requiring the establishment of acetate production first—acetoclastic methanogenesis followed with a delay. In an attempt to gain more insight regarding this question, the effect of spiking the cells with 10 mM of methanogen inhibitor 2-bromoethanosulfonate (2-BES), after acetate production was completely extinguished, was studied (data provided in Figure S3). This led to a sudden—but temporary—recovery of acetate production to similar levels of the earlier peak production, followed by a gradual decline as the inhibitor was depleted by wash-out. The almost direct resumption of acetate production upon spiking with 2-BES could indicate homoacetogens to remain active—even with net production of acetate previously being extinguished—implicating the establishment of a syntrophic interaction between homoacetogens and acetoclastic methanogens in these biocathodes. However, the option of hydrogenotrophic methanogens being responsible for methane production could not be excluded. In the latter, quick resumption of acetate production could be caused by resilient homoacetogens not washed out of the system. Thus, quantitative microbial community analysis would be required to be conclusive towards this point.

**Figure 4:**
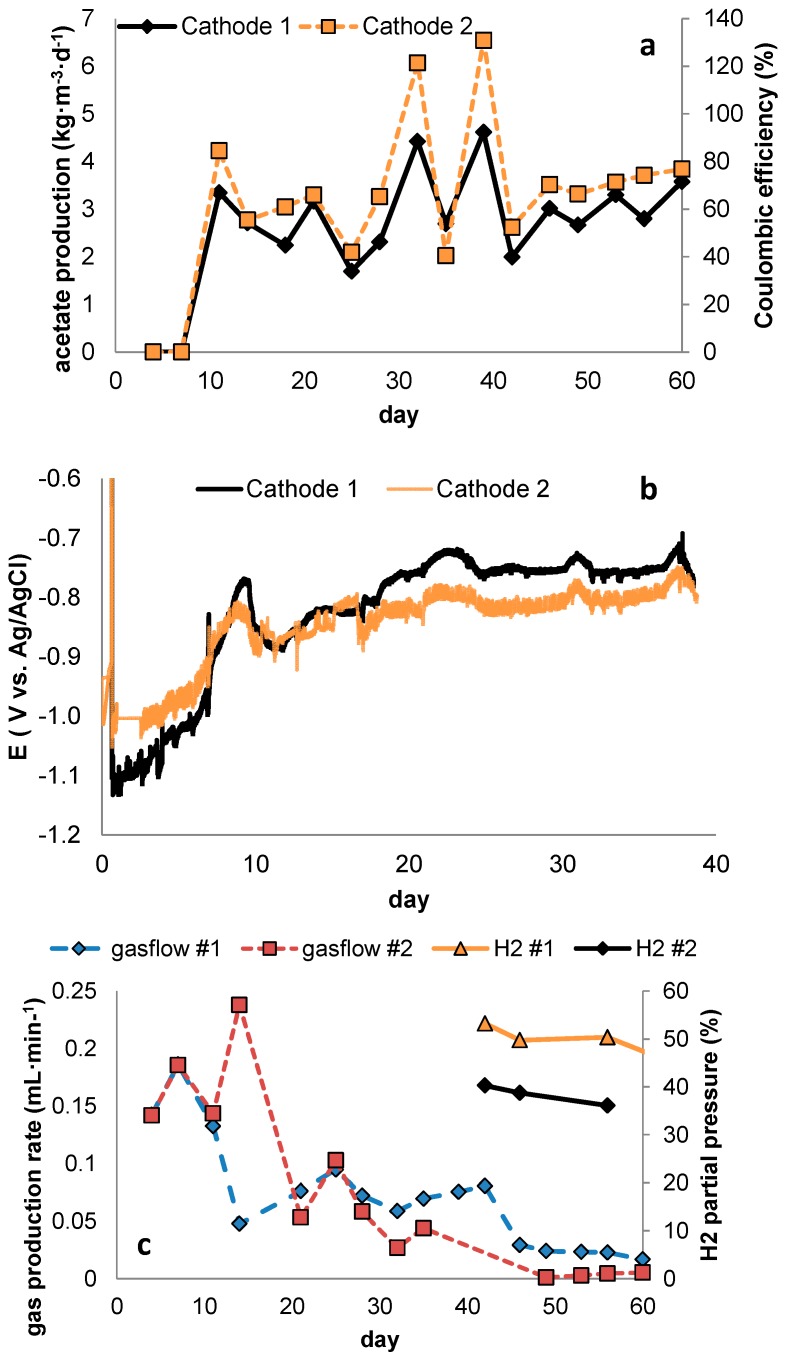
Results obtained in duplicate current controlled cathodes operated at 24 mA (11 A·m^−2^) containing 10 mM of 2-bromoethanosulfonate (2-BES) in the electrolyte; (**a**) Acetate production steadily increased throughout the experimental run of 60 days, reaching nearly 4 kg∙m^−3^∙day^−1^ at a Coulombic efficiency of 80% at the end of the experiment; (**b**) The development of cathode potential over time displayed a steep increase at first, followed by a more stable operation around −800 mV; (**c**) Starting at measurable fluxes, gas production gradually decreased for both cells—as an increasing share of current was directed towards production of acetate—and reached the detection limit by the end of the experiment.

Figure 4 shows the production of acetate in a third round of experiments, in which the highest current density of 11 A·m^−2^ from the second experimental run was applied, but now with continuous addition of 2-BES to the catholyte. When 2-BES was used in combination with this current controlled start-up, long-term stable production of acetate was achieved. Also, in these systems, after prolonged runtime, no visual change in electrolyte turbidity was observed, and measurements of suspended COD showed it to not substantially contribute to the electron balance, strongly suggesting the accumulation of biomass in the porous electrode. Moreover, gas production decreased below measurable fluxes as Coulombic efficiencies towards acetate production increased over time to reach over 80%. The latter showed the ability of the developed biomass to efficiently capture (or prevent the formation of) H_2_, and although hydrogen partial pressures remained around 40%, they still showed a decreasing trend at the end of the experiment. Remarkable was the rapid increase in cathode potential for both cells from around −1100 to −800 mV in the first 10 days, followed by a steady to slightly increasing potential throughout the remaining runtime.

Although hydrogen partial pressures still reached up to 40% at day 40, the observed increase in cathode potential could be explained by enhanced uptake of hydrogen by closely attached biofilm. Apart from hydrogen consumption by acetogenesis affecting product inhibition, the observed increase in cathode potential may also indicate some sort of catalysis, as was previously reported to occur by Jourdin and co-workers [8]. Although the nature of this catalytic phenomenon could not be further elucidated using the current methodology, in their study, Jourdin et al. ascribed the catalysed formation of hydrogen in their system (followed by subsequent production of acetate in case 2-BES is added) to the intracellular biosynthesis of copper nanoparticles, possibly bymethanogens. The reported presence of methanogens under total absence of methane production leads to the hypothesis that these archaea live at the expense of hydrogen catalysis, thus forming an intermediate step during bioelectrochemical acetogenesis rather than a competitive one.

**Figure 5:**
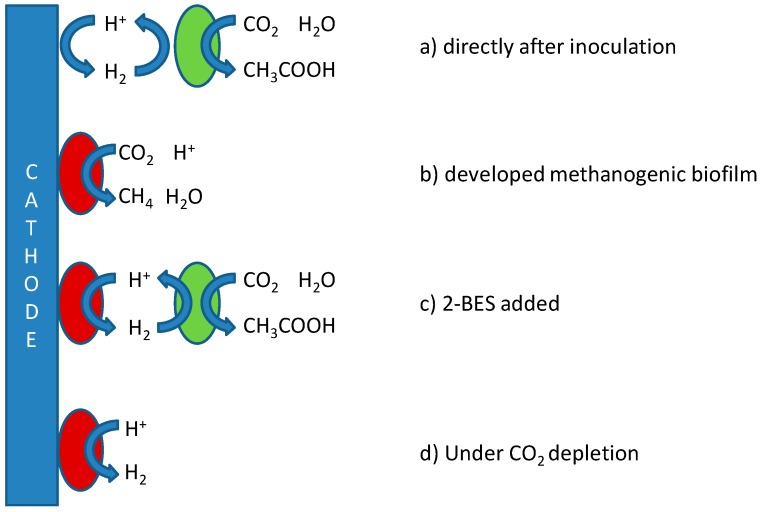
Proposed mechanisms as occurred under tested conditions. Red circles represent methanogenic, and green circles acetogenic bacteria. (**a**) Based on the short start-up required for acetogenesis during current controlled experiments, it is likely that electron transfer during this phase does not require the establishment of a biofilm on the electrode, but is mediated by abiotically formed hydrogen; (**b**) The gradual shift towards production of methane observed during the later phase may be caused by the establishment of a methanogenic biofilm on the electrode, which at that point outcompetes the acetogenic community for the available substrate, rendering the acetogens effectless; (**c**) Upon addition of 2-BES, the methanogenic biofilm redirects electrons to form hydrogen and the produced hydrogen is consecutively used by acetogens to produce acetate; (**d**) In the case of CO_2_ depletion, all electrons are shuttled to the production of hydrogen [8].

Applying this concept of microbe-enhanced hydrogen formation to our results, this would imply that under the conditions tested, an initially acetogen-dominated microbial community was complemented by electrode-bound hydrogen producers, possibly methanogens, which initially only directed part of the current to methane. As the concentration of these bacteria increased, so did the capacity for methane formation, eventually resulting in the extinction of acetogenesis as less and less current was turned into hydrogen by the methanogens. A schematic overview of these proposed dynamics, which would need community analysis or Fluorescent In-situ Hybridization (FISH) assisted imaging to be confirmed, is depicted in Figure 5.

## 3. Discussion

A well-working acetate producing MES (1) has high product selectivity; (2) has high stability/robustness against contamination and/or washout; and (3) does not require costive and/or toxic additives (e.g., 2-BES). In addition, from an application perspective, attention should be paid to: (1) obtaining high product concentrations; (2) high volume specific reaction rates, resulting in smaller reactor footprints; and (3) high energy efficiency, producing the desired compound at the lowest energy expenses.

In the presented research, the effect of different electrochemical control modes on both start-up and steady-state characteristics was investigated. Here, the obtained results will be discussed in relation to abovementioned aspects/criteria.

### 3.1. Current Density as Control Parameter for MES Selectivity towards Acetate

From the perspective of reaction specificity, methanogen abundance needs to be kept as low as possible. To achieve this, reactor conditions should be so that in the steady state established, achieved specific growth rates for acetogenic organisms are as high as possible when compared to those of methanogens. For the sake of brevity, in the discussion that follows no distinction is made between hydrogenotrophic methanogenesis and homoacetogenesis followed by acetoclastic methanogenesis, as the overall reaction is identical for both.

**Figure 6:**
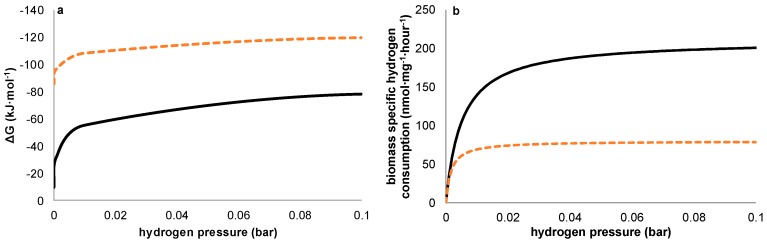
Shows the effect of variable hydrogen pressures on the competition between acetogens and methanogens both in terms of (**a**) Gibbs free energy of reaction [12]; and (**b**) biomass specific substrate consumption rate (Monod parameters for modelling growth were taken from reported values in Kotsyurbenko et al. [21]). As can be seen, methanogens have a thermodynamic advantage over homoacetogens throughout the whole domain. This thermodynamic advantage is reflected in the growth curves through the hydrogen affinity (*K_s_*) for methanogens in general being lower than for acetogens. However, acetogenesis achieves higher conversion rates at higher hydrogen partial pressures (µ_max_) compared to methanogenesis, rendering hydrogen pressure an essential parameter to steer competition between acetogens and methanogens [21].

In none of the experiments presented here in which no 2-BES was added was a steady-state achieved in which measurable quantities of acetate were produced. The experimental conditions were thus rendered ineffective in outcompeting methanogens sufficiently during steady state reactor operation. However, during the current controlled experiments, acetate production was positively related to current density. In potential controlled experiments, net acetate production only occurred when current increased rapidly, thus clearly not reflecting a steady state. As was already stated in the introduction (Table 1), from a thermodynamic perspective, methanogens can gain more energy than acetogens from the consumption of hydrogen and CO_2_. Figure 6a shows this thermodynamic advantage for methanogens is valid at any hydrogen pressure. Our observations however suggest that—under the conditions chosen—a kinetic difference exists between the acetogens and methanogens, both present in the used inoculum, resulting in a higher net growth rate for acetogens during start-up despite the higher thermodynamic available energy for methanogens. Although theoretically, an over-representation of acetogenic organisms in the used inoculum could have resulted in similar results without the requirement of higher growth rates for acetogens, the observations on the potentiostatic experiment with a prolonged period of methanogenic activity prior to acetogenesis strongly indicates this not being the case. Further supporting our hypothesis, differences in growth kinetics have been described previously for methanogens and acetogens using hydrogen as common electron donor [20,21]. To allow comparison between thermodynamics and kinetics, Figure 6a,b illustrates both the thermodynamic equilibria and biomass specific substrate consumption rates which result at different hydrogen pressures. As substrate consumption is directly related to growth via yield, this shows homoacetogens thrive better than methanogens at higher hydrogen partial pressures, despite the higher potential energy gain available to methanogens.

As in our experiments the production of acetate was always accompanied by elevated hydrogen partial pressures, we presume electron transport from electrode to acetate to be primarily mediated through hydrogen. As such, the relative growth rates of both methanogens and acetogens were directly affected by the hydrogen partial pressure, in turn resulting from the fluxes obtained by, on the one hand, production of hydrogen by the set current density and, on the other hand, its consumption by microbial activity. At this point, little is known about the relationship between current density and local hydrogen partial pressures at the electrode, and this is likely to be specific for different electrode materials, depending on how accessible the material is to biofilm formation (e.g., hydrophobicity, roughness, decorated by functional groups etc.). Therefore, rather than predicting an optimal current density based on reported growth parameters, a more empirical approach towards optimization of current density with relation to biocathode selectivity will be required. This is the case both when hydrogen shows an inevitable intermediate in biocathodic production of acetate—as too high current density will result in loss of selectivity by evading hydrogen gas—and in the case alternative mechanisms turn out to play a role in electron transfer from electrode to microbe. The uptake of molecular hydrogen by both acetogens and methanogens is mediated by ferredoxin reducing hydrogenases [22,23], a group of enzymes known to possess typically high turnover rates [24]. Given the mechanistic similarities and characteristics occurring at the start of both pathways [25,26], the kinetic advantage of acetogens compared to methanogens at higher hydrogen pressures is probably due to a conversion further down the electron transfer chain (ETC) of methanogens becoming rate limiting under these conditions. In contrast, at low hydrogen pressures, the first electron step (thus from hydrogen to the ETCs initial electron acceptor) in acetogens is likely to be thermodynamically controlled (instead of kinetically). The observation that the rate-limiting step for methane formation occurs further down the ETC is of importance, as it makes it more likely that this limitation will remain in case of an alternative mechanism of electron transfer between cathode and microbe. This would give the homoacetogens a competitive advantage over methanogens at higher current densities regardless of the exact nature of electron transfer between electrode and microbe. Therefore, we hypothesize current density to be a defining parameter for selectivity in acetate-producing MES. At the same time it should be noted that in the presented data current densities were probably not high enough to establish long term stable production of acetate.

### 3.2. Considerations on the Effect of Current Density on Overall Reactor Performance

As previously stated, selectivity is not the only criterion defining reactor performance in MES, and it therefore holds relevance to further delineate the effects current density presumably has on product concentration, reaction rates and energy efficiency.

In terms of volume specific reaction rates, the application of high current densities is attractive as it drives acetogens close to their maximum turnover rates. However, to sustain production at high current densities, high biomass densities will be required as well. To achieve this, assuming that a biofilm forms on the electrode surface, it becomes crucial to use an electrode material with high specific surface area (with pores accessible to microorganisms).

In case of biofilm formation, the solid/hydraulic retention time ratio (SRT/HRT) can be tuned e.g., by inducing various levels of sheer stress on the biofilm, for instance by recirculation at different flowrates, and an optimum with respect to process selectivity might be sought. To this end, one has to bear in mind that a trade-off will show between biomass retention time and product selectivity, as also methanogens will be washed out slower and may win competition if conditions are suitable (Figure 6).

Inherent to higher current densities is also the occurrence of higher overpotential, and although the use of high specific surface area electrode materials may lower this, this will lead to lower voltage efficiency. At the same time, higher current density presumably leads to higher Coulombic efficiency towards acetate (through improved selectivity). Thus, a compromise has to be found between selectivity and voltage efficiency to obtain optimal overall energy efficiency.

To summarize, we hypothesize that, in order for an acetate-producing cathode to perform well both in terms of product selectivity, production rate and obtained product concentrations, a high current density will have to be combined with a high specific surface area electrode, allowing a (relatively) high biomass density at a (relatively) low biomass retention time.

## 4. Materials and Methods

### 4.1. System Design and Reactor Assembly

Experiments were conducted in electrochemical cells consisting of two plexiglass flow compartments, two flat plate electrodes and two plexiglass support plates as described previously [27]. The assembly had an effective projected electrode surface area of 22 cm^2^ for both the cathode and anode and an effective cathode volume of 33 cm^3^. Cathodes consisted of a stainless steel (SS316) plate serving as current collector, with a plain sheet of graphite paper and five layers of graphite felt (thickness 3 mm, FMI Composites Ltd., Biddeford, Scotland, UK) firmly held between current collector and membrane, thus with the felt serving as the actual electrode material in contact with the electrolyte. Anodes consisted of flat platinum/iridium coated titanium plates (Pt/IrO_2_ 80:20, Magneto special anodes BV, Schiedam, The Netherlands) with the anode flow compartment filled with polypropylene spacer material sandwiched between the anode and membrane, ensuring proper flow distribution and providing support to the membrane. An Ag/AgCl reference electrode (model QM711X/Gel, Prosense, Oosterhout, The Netherlands) was connected to the cathode using a Haber-Lugging capillary filled with saturated 3 M KCl (+0.205 V vs. NHE). All potentials reported were measured relative to this reference. The anode and the cathode compartments were separated by a cation exchange membrane (Ralex™ C(M)H-PES, Vysočany, Czech Republic). Total catholyte recirculation volume was 180 mL, anolyte recirculation volume 120 mL. Electrolytes were recirculated at a pump speed of 80 mL∙min^−1^. In-line pH/temperature monitoring was performed on the catholyte (Endress + Hauser, sensor model CP571D-7BV21 connected to a Liquiline datalogger). Temperature of reactors was maintained constant at 32 ± 1 °C using climate control of the research cabinet, and systems were shielded from light to avoid the possibility of photoautotrophic activities during the experiments. An Ivium N-stat multichannel DC potentiostat (Eindhoven, The Netherlands) was used in a three-electrode configuration (working, counter, and reference) to perform electrochemical measurements and experiments. Recording and analysis of potentiostat data was carried out using the Iviumstat software (Iviumsoft 2.693, ivium, Eindhoven, The Netherlands).

### 4.2. Electrolyte Composition

Catholyte was made using MilliQ water containing 0.4 g∙L^−1^ NH_4_HCO_3_, 0.05 g∙L^−1^ Ca(OH)_2_, 0.1 g∙L^−1^ MgSO_4_∙7H_2_O, 0.87 g∙L^−1^ K_2_HPO_4_, 0.68 g∙L^−1^ KH_2_PO_4_, 1 mL∙L^−1^ of vitamin solution [3] and 1 mL∙L^−1^ of trace metal solution [28]. In order to ensure anaerobic conditions and high concentrations of CO_2_ in the influent, the medium was continuously purged with CO_2_ prior to injection. Using this practice, a total inorganic carbon concentration (TIC) of 11 mM was dissolved in the catholyte, buffering the pH of the catholyte at 5.5.

In later current controlled experiments, the sodium salt of the methanogen inhibitor 2-bromoethanosulfonate (2-BES) was added in order to suppress methanogenic activities at a concentration of 2.1 g∙L^−1^. Anolyte consisted of a 10 mM phosphate buffer adjusted to pH 7 before use. Anolytes were sparged continuously with CO_2_ to remove any formed oxygen throughout the experiments while preventing cross-membrane CO_2_ stripping.

### 4.3. Inoculum

A mixture of activated sludge from an anaerobic digester and cow manure was used as a source of microorganisms. Homogenized activated sludge and cow manure were centrifuged separately for 5 min at 3700 rpm and supernatant from both solutions were mixed in a 2:1 ratio, resulting in the final inoculum with a COD of 12.5 g∙L^−1^. Upon inoculation, each cell was injected with 10 mL of inoculum leading to an initial COD of 0.7 g∙L^−1^ in the catholyte at the start of biotic experiments.

### 4.4. Hydraulic and Electrochemical Operational Conditions

Systems were filled and continuously fed with fresh electrolyte giving an HRT of 40 h (potentiostatic experiments) or 20 h (current controlled experiments). Prior to inoculation, potentials/currents were applied for at least 2 days until potentials/currents stabilized, thus serving as baseline. In the first run, four cells were poised each at a different potential. Based on the equilibrium potentials for formation of hydrogen and acetate from CO_2_, the following four potentials were chosen:

The first was −560 mV; the standard equilibrium potential of direct carbon dioxide reduction into acetate is −490 mV. The first setup aimed at facilitating this reaction by imposing only a small overpotential of 70 mV. Doing so, the formation of hydrogen gas is thermodynamically unfeasible and alternative electron transfer is therefore required. This may imply direct electron transfer, providing no other electron mediators are present in the system.

The second potential was −630 mV; selected based on the same rationale as for setup 1, but with a slightly increased overpotential.

The third potential was −700 mV; at this potential, hydrogen formation in theory becomes feasible but due to large overpotentials associated with hydrogen formation a catalyst would be required.

The fourth potential was −900 mV; well below the equilibrium potential for hydrogen formation in these systems, a small cathodic current of 2–4 mA was observed during the abiotic operation of the system, providing sufficient reductants at start.

During the potential controlled experiments, systems were put in batch mode by stopping influent pumps directly after inoculation for 72 h to prevent washout. After this initial batch phase, influents were resumed at the set HRT of 40 h.

During the current controlled experiments all cells were set to an HRT of 20 h, thereby allowing a fixed and equal flux of CO_2_ to all cathodes. HRT was maintained also directly after inoculation, as preliminary results showed good retention of biomass in the graphite felt even after prolonged periods of inactivity and continuous flushing.

### 4.5. Analytical Techniques

Liquid and head space samples (1 mL) were taken from catholytes, once a week or more frequently when observed changes in currents/potentials or gas fluxes justified this. Occasionally, the anolyte was sampled for its ionic composition to assess crossover rates of ions. TIC/TOC measurements were done by using a Shimadzu TIC/TOC analyser (Kyoto, Japan); model TOC-VCPH in combination with Shimadzu ASI-V Autosampler (Kyoto, Japan). Gas samples were taken from the headspace in the cathode chamber and analysed for H_2_, CO_2_, CH_4_ and O_2_ using a gas chromatograph (Varian CP-4900 microGC, TCD detector, Mol Sieve 5A PLOT and PoraPlot U columns in parallel, Santa Clara, California, USA). The catholyte COD was determined using a spectrophotometer HACH XION 500 (HACH, Loveland, CO, USA) with test kit (No. 414, Hach-Lange, Tiel, The Netherlands). For obtaining estimates on biomass concentrations, the COD of both unfiltered and filtered (0.45 µm) electrolytes were compared. HPLC analyses for detection of organic acids (formic acid, acetic acid, propionic acid and butyric acid) were performed with a Dionex UHPLC system (Waltham, MA, USA).

### 4.6. Performance Calculations

With acetate being the target product, Coulombic efficiency (CE) expresses the fraction of the electron flow (which is dictated by the current) which is used to form acetate:

$$CE=\frac{nF\phi \Delta c_{{\mathrm{Ac}}^{-}}}{I}$$

where *F* is Faraday’s constant (96,485 C∙mol^−1^), Δ*c*_Ac_− is the difference in acetate concentration in the effluent (mol∙L^−1^) as compared with the influent, φ is the total volumetric flow of influent (L∙s^−1^), *n* is the number of electrons consumed per mol acetate produced (8), and *I* (A) is the steady state electrical current.

## 5. Conclusions

We propose current density as a most promising operational parameter for optimizing acetate producing biocathodes. Future experimental efforts should be directed towards a more quantitative investigation of current density and its relation to reactor performance. Therefore, in our opinion, acetate-producing cathodes may be better controlled at a set current, rather than at a controlled potential. The here presented data shows that start-up strategy is important for reactor performance in acetate producing cathodes. Within the experimental periods tested, controlled current showed to be more successful than controlled potential in establishing the production of acetate by reduction of CO_2_, although it should be noted that with the current densities tested, no long-term stable acetate production was established. We therefore propose to test higher current densities in future experiments. Closely related to current density, a primary role is foreseen regarding the used electrode materials, as it forms the most important design variable in a biocathode and determines to a large extent—amongst other aspects—biomass attachment/retention specifics and electrode specific surface area. We argue that by optimizing reactor control by these means, neither extensive pre-enrichment of biota nor selective inhibition of methanogenesis by means of 2-BES is required to obtain selective acetate production at high reaction rates and concentrations in the future.

## Figures and Tables

**Table 1:** Overview of reactions involved in methanogenesis and homoacetogenesis by reduction of CO_2_. Depicted are both half reactions (upper) and overall reactions using hydrogen or acetate as an electron donor. Standard reaction energies/potentials were taken from Thauer et al. [12] and expressed at pH 7, 298 K with all other reactants at standard concentrations.

Process	Reaction	*E*’^0^/V (vs. Ag/AgCl)	
Half reactions			
Methanogenesis	CO_2_ + 8e^−^ + 8H^+^ ⇌ CH_4_ + 2H_2_O	−0.24 V	
Acetogenesis	2CO_2_ + 8e^−^ +8H^+^ ⇌ CH_3_COOH + 2H_2_O	−0.29 V	
Hydrogen oxidation	2H^+^ + 2e^−^ ⇌ H_2_	−0.41 V	
Overall reactions in anaerobic digestion			ΔG_r_ (kJ∙mol^−1^)
Homoacetogenesis	4H_2_ + 2CO_2_ ⇌ CH_3_COOH + 2H_2_O	0.12 V	−95
Hydrogenotrophic methanogenesis	4H_2_ + CO_2_ ⇌ CH_4_ + 2H_2_O	0.17 V	−131
Acetoclastic methanogenesis	CH_3_COOH ⇌ CH_4_ + CO_2_	0.05 V	−36

## Supplementary Materials

Supplementary materials can be found at www.mdpi.com/1422-0067/18/1/204/s1.

## Abbreviations

MES	Microbial Electrosynthesis
BES	BioElectrochemical System
2-BES	2-BromoEthanoSulfonate (sodium salt)
ETC	Electron Transfer Chain
