# Supplementary material for: Competition between Methanogens and Acetogens in Biocathodes: A Comparison between Potentiostatic and Galvanostatic Control
Source: Int J Mol Sci. 2017 Jan 19;18(1):204. doi: 10.3390/ijms18010204 (PMC5297834; doi:10.3390/ijms18010204)
Supplement: Supplementary file 1 [file ijms-18-00204-s001.pdf]

Supplementary Materials: Competition between Methanogens and Acetogens in Biocathodes: A Comparison between Potentiostatic and Galvanostatic Control

Sam D. Molenaar, Pradip Saha, Annemerel R. Mol, Tom H. J. A. Sleutels, Annemiek ter Heijne and Cees J. N. Buisman

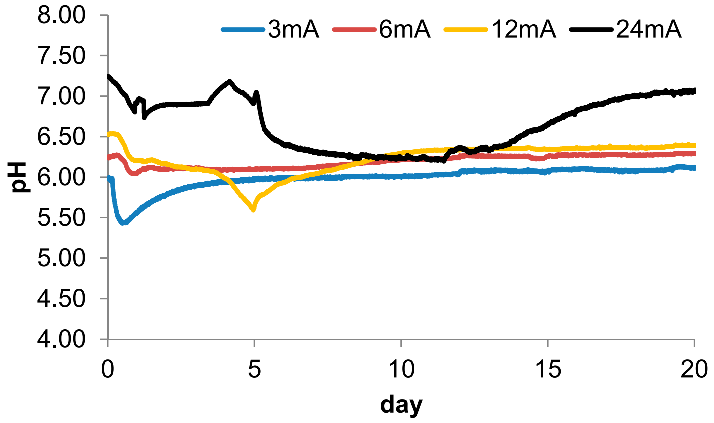

Figure S1. pH profiles of the 4 different current densities tested during the current controlled experiments.

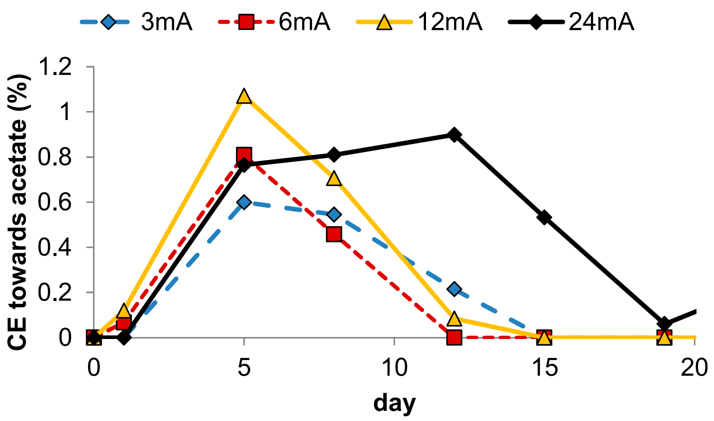

Figure S2. Acetate productions of the 4 different current densities tested during the current controlled experiments, with acetate production expressed as Coulombic efficiency (%), so normalized to the applied current.

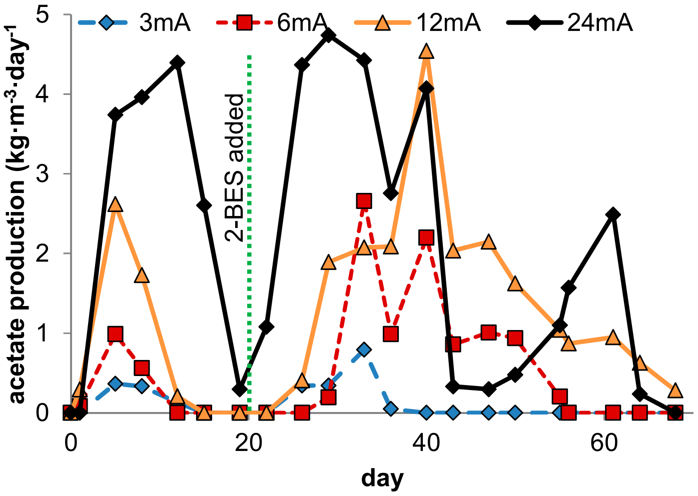

Figure S3. Acetate production rates as measured throughout the extended runs of the 4 current densities tested during the current controlled experiments. The green dotted line indicates the moment the systems were spiked with 10 mM of the methanogen inhibiting compound 2-bromoethanosulfonic acid (2-BESA).
